# Bar-HRM for authenticating soursop (*Annona muricata*) tea

**DOI:** 10.1038/s41598-018-31127-9

**Published:** 2018-08-23

**Authors:** Maslin Osathanunkul

**Affiliations:** 1 Department of Biology, Faculty of Science, Chiang Mai University, Chiang Mai, Thailand; 2 Center of Excellence in Bioresources for Agriculture, Industry and Medicine, Chiang Mai University, Chiang Mai, Thailand

## Abstract

Drinking soursop (*Annona muricata*) tea has become popular in Thailand due to recent findings about the medicinal properties of soursop tea regarding anti-cancer in particular. Consequently, numerous *A. muricata* tea products were found to be sold on markets and relatively expensive. It is almost impossible to identify the plant species component in the tea bag or powder products using traditional methods which are based on morphological characters. Therefore, a main objective of this study is to develop a molecular method called Bar-HRM (DNA barcoding coupled with High Resolution Melting) for authenticating *A. muricata* products. Three chloroplast regions including *matK*, *rbcL* and *trnL* were selected for *in silico* analyses. The findings show that *rbcL* is the most suitable region to be used for species identification in HRM analysis. Eleven *A. muricata* herbal products were purchased and tested with *rbcL* primers. Results from melting profile indicated that three out of eleven tested products were adulterated with other *Annona* species. It is believed that the *Annona* products are adulterated to increase the quantity and to make more profit. Notably, all of the tested products purchased from local producers were found to contain herbal species that differ from the species indicated by the seller.

## Introduction

Traditional medicines have played an important role in human healthcare for thousands of years and are still widely used today. Medicinal plants have been the basis for traditional remedies, a source of nutrition around the world^1^. Plants with medical potentials have been used as alternative medicines for various treatments of diseases in both developed and developing countries. Thus, pharmacological studies about traditional herbal medicine are now a critical part in global health. This is not to mention that around 80% of current drugs used are related to the active compounds of the medicinal plants^2–5^. It is therefore, unsurprising that traditional herbal medicines are getting significant attention worldwide. However, as attention and research funding for international traditional herbal medicine grows, the safety of the global herbal market should not be overlooked. The majority of alternative medicines currently available on the market are in processed form which are derived from natural products, mainly from higher plants. Depending on the particular country and existing legislation, herbal products are normally regulated. The standard for the assessment of herbal medicines was set and proposed by several organisations with accessible guidelines^6,7^. A large number of counterfeiting and adulteration in herbal products was reported. The perception that herbal drugs are very safe and free from side effects is not the reality. Generally, plants have hundreds of constituents and some are very toxic. Taking the wrong herbal drugs could have adverse effects.

*Annona muricata* is a member of the Annonaceae. It is known as soursop with Thai native vernacular name as ‘Thu-Rein-Thed’. *A. muricata* is a tropical and subtropical plant species known for its edible fruit which used in several different ways such as in the field of culinary (e.g. syrup, ice cream, candies) and particularly medical treatments^8,9^. Based on numerous pharmacological studies, the *A. muricata* was found to possess anti-cancer, anti-tumor, anti-bacterial, anti-inflammatory, anti-parasitic, anti-malarial and anti-ulcer activities^10–12^. Recently, *A. muricata* has become increasingly popular due to its anti-cancer properties. From 2012 to 2014, cancer overtook heart disease as the main cause of death in Thailand with huge increase each year^13^. Potential studies of *A. muricata* on cancer curing were not only limited to *in vitro* and *in vivo* evaluations. Consumption of boiled water infusion of the *A. muricata* leaves resulted a stabilisation of the metastatic breast cancer in a case study of a 66 year old woman for 5 year^14^. Herbal tea is a beverage brewed from dried leaves, seeds, flowers, stems or roots of plants species rather than *Camellia sinensis*, which has been gaining popularity in Thailand. *A. muricata* is now made into a tea due to the promising anti-cancer and anti-tumor activity. There is inevitable adulteration when the demand and prices increase and thus, reliable quality control methods for medicinal plant materials become necessary.

DNA barcoding is a method using a short fragment of DNA (~500 base pair) for species identification and taxonomic classification. DNA barcodes have been successfully used in animal species identification using cytochrome c oxidase subunit I (COI) in mitochondrial genome^15–17^. Regions in the chloroplast and nuclear genome were recommended in plant including coding regions (*rbcL* and *matK*) and non-coding regions (*trnL* and *trnH*-*psbA* in chloroplast genome and internal transcribed spacers in nuclear genome)^18,19^. The DNA barcoding approach has been applied to use for detection of adulteration in food and herbal products with success^20–22^. However, some limitations of the method mean it is not fully practical in some developing countries like Thailand. Main drawbacks are likely to be that it is time-consuming and its high costs due to outsourced sequencing. Recently, DNA barcoding has been applied to be used in combination with High Resolution Melting analysis, called Bar-HRM. The Bar-HRM was proved to be a good compromise for counterfeit herbal products and adulteration detection. The Bar-HRM is a sequencing free method using fluorescent dye for detection of double-stranded DNA in real-time PCR. Increasing temperature during the process lead to denaturation of double-stranded DNA into single-stranded DNA and melting temperature (T_m_) is measured. The Bar-HRM analysis is not only rapid, cheap (in long term and large scale investigation), and feasible for accurately species discrimination in plants^23–27^.

In consequence, the Bar-HRM is one of many promising techniques for counterfeit and adulterant detection in soursop commercial products sold on markets in developing country like Thailand. The majority of soursop products are very expensive and available in tea bags. Here, the replacement of *A. muricata* with lower grade or cheaper substitutes such as custard apple leaves (*Annona squamosa*) and jackfruits leaves (*Artocarpus heterophyllus*) in commercial products was investigated. In addition, this work evaluated three chloroplast regions including *matK*, *rbcL* and *trnL* to find out which DNA barcode region is the most suitable for identifying the three plants species (*A. muricata, A. squamosal* and *A. heterophyllus)*.

## Results and Discussion

### Data mining and *in silico* analyses

**Figure 1:**
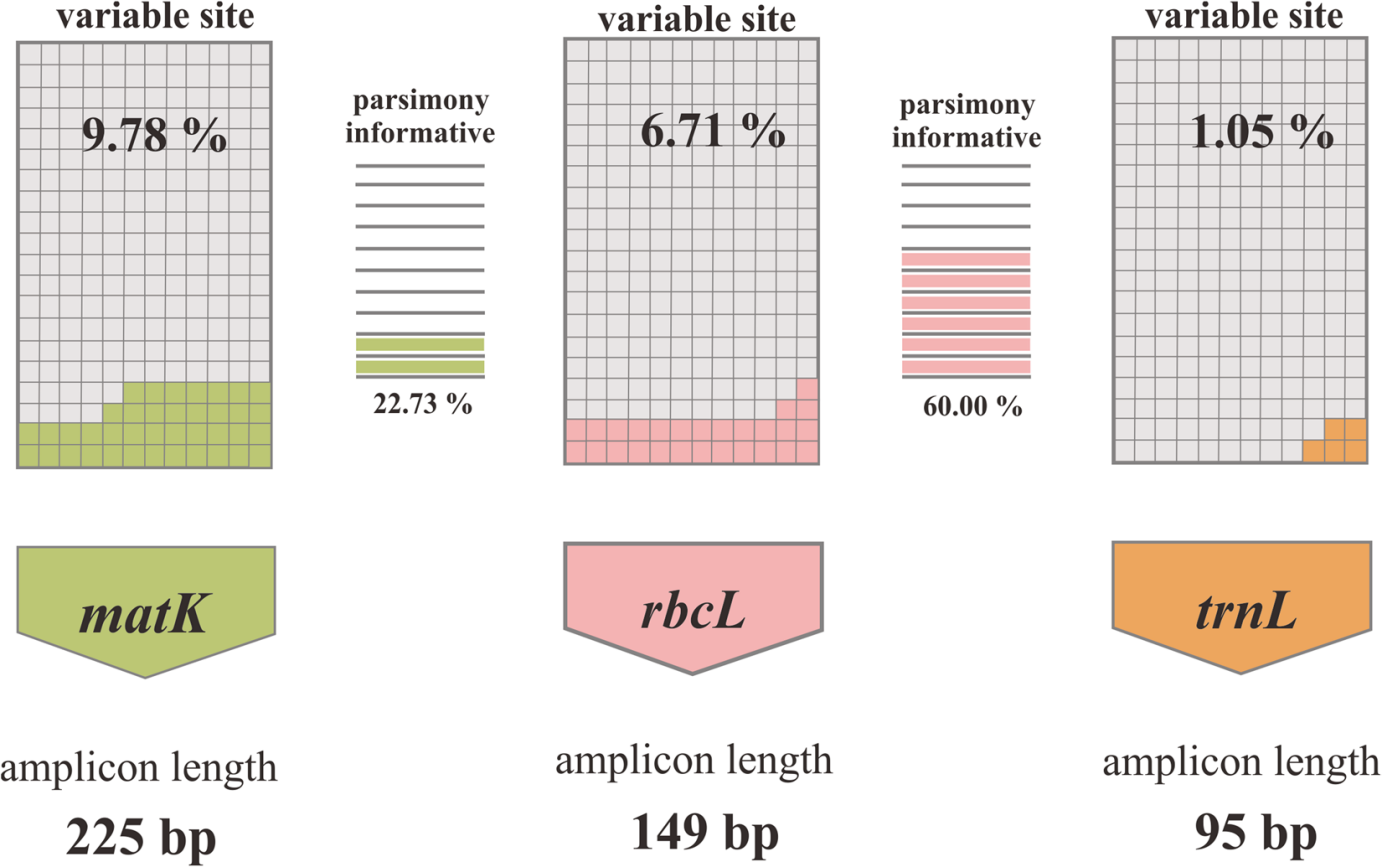
Variable sites found in each analysed region (*matK, rbcL* and *trnL*) and parsimony-informative type of *matK* and *rbcL* amplicons.

The number of DNA sequence records of *Annona* species retrieved from GenBank of each barcode region are 16, 25 and 12 species for *matK, rbcL* and *trnL*, respectively. All retrieved sequences were then analysed using MEGA 6 program for conserved site (%), variable site (%), parsimony-informative site (%), singleton site (%), conserved forward primer/total (%) and conserved reverse primer/total (%). The longest and shortest sequence of each region was found to be 566–1,763 bp of *matK*, 550–1,469 of *rbcL* and 864–947 for *trnL* and after filtering and trimming the analysed sequences length were reduced to 507, 466 and 873 bp of *matK, rbcL* and *trnL*, respectively. As can be seen from Table 1, the analysed *matK* fragment was observed to have higher nucleotide variation (9.78%) than both *rbcL* (6.71%) and *trnL* (1.05%). It is indicated that *trnL* is least suitable to be used for our tested species identification whereas *matK* is likely to be the best region for this study. However, further investigation revealed that *matK* was not as good as *rbcL* region for HRM analysis. Although the analysis of *matK* sequences showed higher number of nucleotide variation than that found in *rbcL*, most of variable sites of the *rbcL* are parsimony-informative type (Fig. 1).

**Table 1:** Characteristics records from *in silico* analysis of each region.

Markers	*matK*	*rbcL*	*trnL*
Available species	16	25	12
Shortest and longest sequence (bp)	566–1,763	550–1,469	864–947
Characters used in analysis (bp)	507	466	873
Product size (bp)	225	149	95
Variable site/total (%)	22/225 (9.78)	10/149 (6.71)	1/95 (1.05)
Parsimony-informative site/total (%)	5/22 (22.73)	6/10 (60.00)	1/100 (100)
Singleton site/total (%)	17/22 (77.27)	4/10 (40.00)	0/1 (0.00)

**Figure 2:**
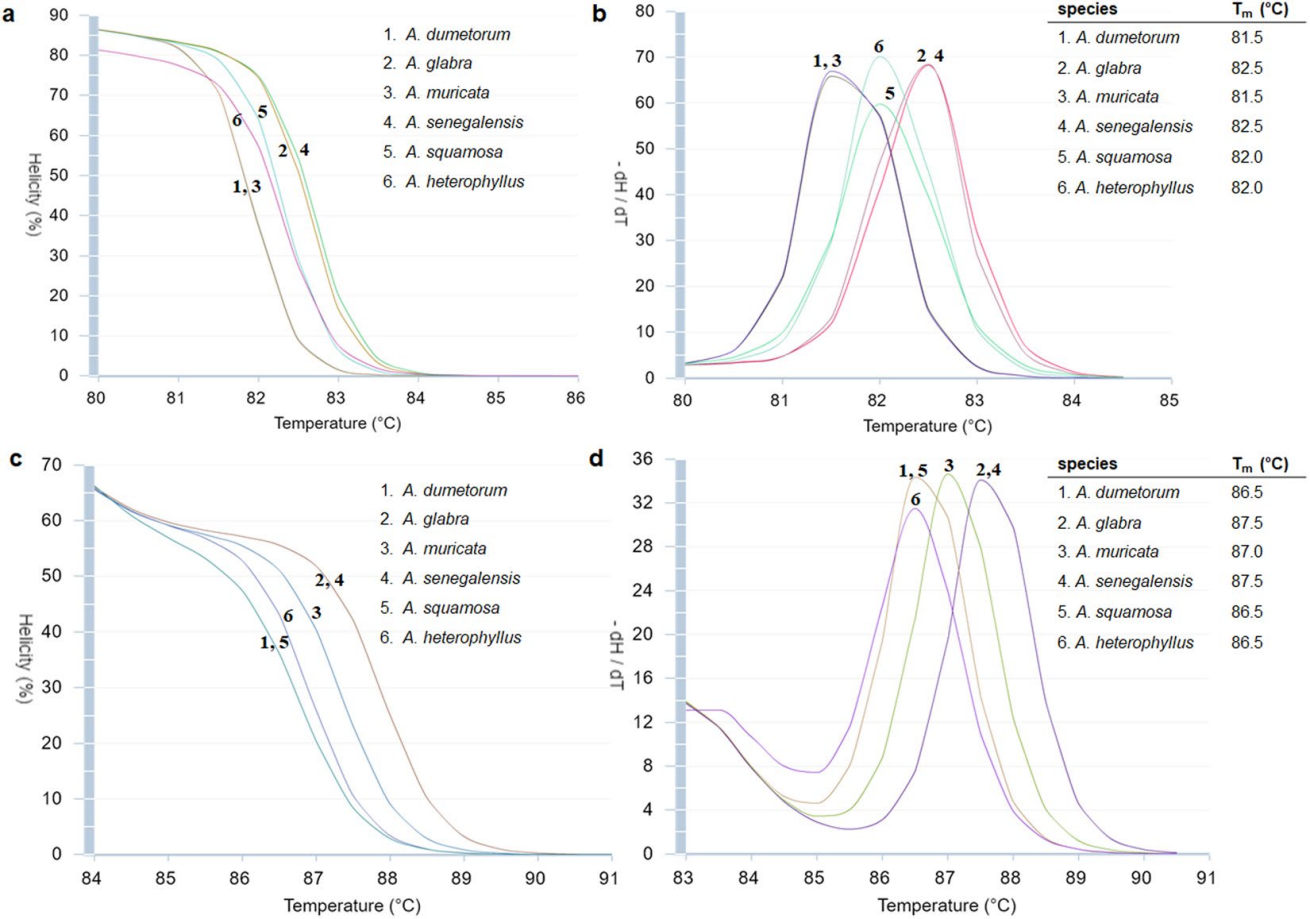
Normalised curves and Tm generated from simulated HRM analyses (uMeltSM). (**a**) and (**b**) *matK* primers, (**c**) and (**d**) *rbcL* primers with sequences from six species included *A. dumetorum*, *A. glabra*, *A. muricata*, *A. senegalensis*, *A. squamosa* and *A. heterophyllus*.

Thus, interspecific variation of *matK* are lower than *rbcL* for discrimination of *Annona* species. In addition, our target species (*A. muricata*) could be discriminated from other tested species in simulated HRM (uMelt^SM^) only by *rbcL* marker not *matK* (Fig. 2a–d). The different barcode regions tend to work well in different plant groups so the choice of barcode used in each experiment depends on the plant group studied^28^. Previously, the *rbcL* had been used for *Annona* species identification by the number of studies, although some suggested the use of both *matK* and *rbcL*^29,30^. Primer pairs should yield an amplicon of 100 to 300 bp for effective HRM analysis^31^. In this study, amplicon products derived from *rbcL* primers were found to be 149 bp in length which is suitable for HRM analysis.

### Real-time PCR for high resolution melting (HRM) analysis

**Figure 3:**
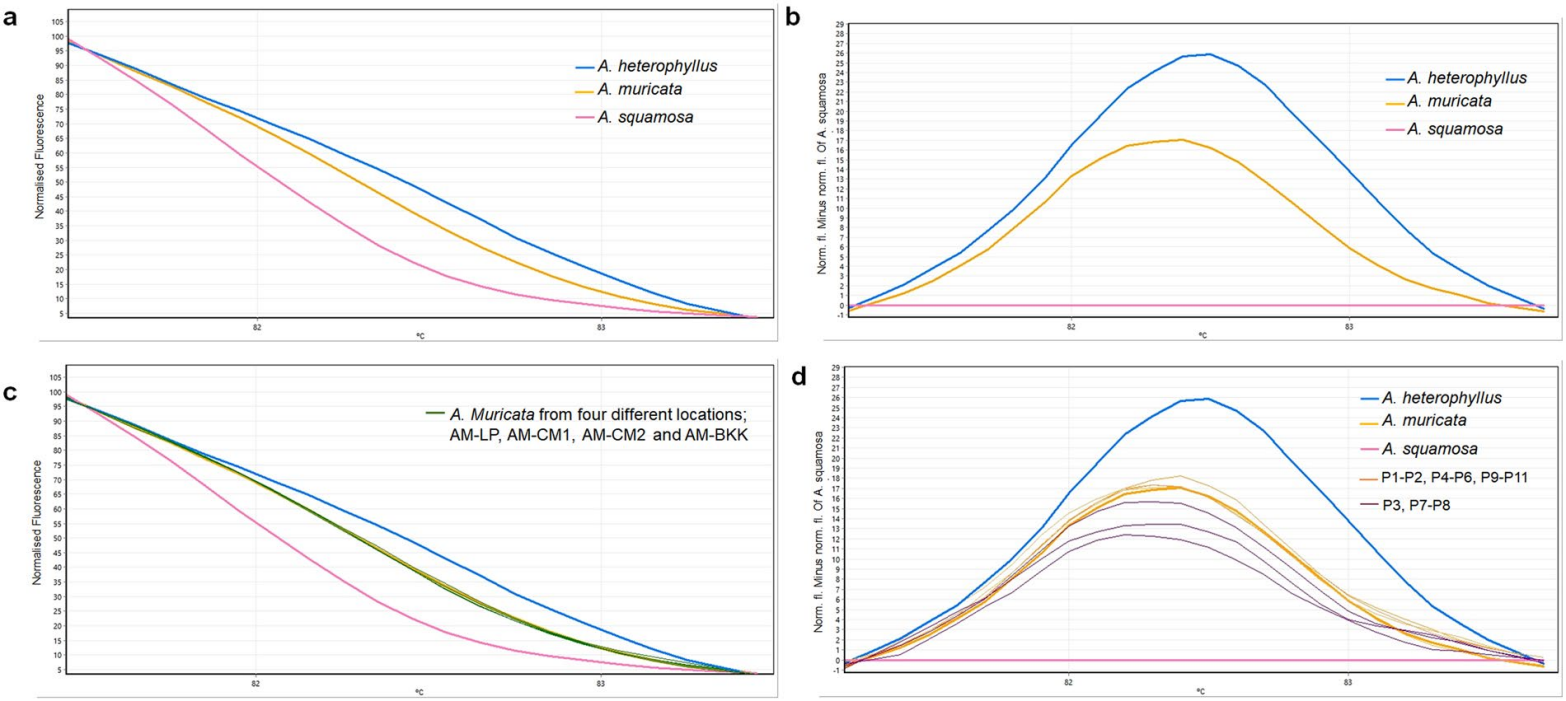
Melting curve profiles of amplicons obtained from *rbcL* primers (**a**) Normalised melting curves, (**b**) Difference melting curves from the three reference species plants, (**c**) Melting curves of *A. muricata* collected from four locations (AM-LP, AM-CM1, AM-CM2 and AM-BKK) compared with the reference curves, (**d**) Difference melting curves of all eleven tested products compared with the three reference species plants.

The real-time PCR and HRM analysis result of the *rbcL* is shown as melting curves. This indicates the *rbcL* has an adequate ability to be used for discriminating all the three reference plant species (*A. muricata*, *A. squamosa* and *A. heterophyllus*) (Fig. 3a,b). The melting curves of the three tested species were clearly separated whereas the curves of samples from same species even collecting from different location are identical (Fig. 3c). Melting temperatures (T_m_) of *A. muricata*, *A. squamosa* and *A. heterophyllus* were 82.36 ± 0.06, 81.86 ± 0.03 and 82.58 ± 0.04, respectively. Genotype confidence percentage (GCP) values were calculated, and a cut-off value of 90% was used to assign a genotype. A cut off value for all three genotype based on SD subtracted from the mean GCP were as followed; 95.15 for *A. muricata*, 94.31 for *A. squamosal* and 98.01 for *A. heterophyllus*. Therefore, the melting curves of the three tested species were then used as references in adulteration test.

An investigation of substitution or adulteration in eleven soursop tea commercials products was then carried out. Real-time PCR and HRM analysis using the *rbcL* primers revealed that substitution or adulteration occurred in three out of the eleven tested products (Fig. 3d). The melting curves of the three (P3, P7 and P8) were shifted from the curve of pure *A. muricata* DNA. The curves were close to the curves of *A. squamosa* DNA, so it is expected that the substitution or adulteration material would be the *A. squamosa*.

**Figure 4:**
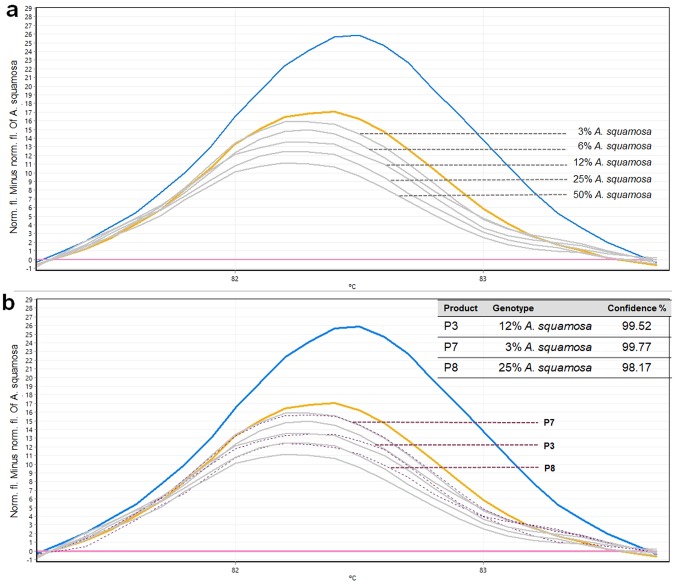
Melting curves obtained by high resolution melting analysis of the mixtures of *A. muricata* and *A. squamosa*, (**a**) Specific amplicons and applied to reference mixtures containing 6, 12, 25 and 50% of *A. squamosa* in *A. muricata*, (**b**) Difference curves of the three tested products comparing with the melting profiles of the mixtures.

Detecting adulteration in *A. muricata* products was then tested using the developed method. The results of the validation method with *A. muricata* spiked with *A. squamosa* in different proportions (6%, 12%, 25% and 50%) were shown in Fig. 4a. These results show the analysis for one experiment as all three experiments gave similar results, thus showing very good reproducibility. As expected, the level of contamination resulting from adulteration alters the shape and shifts proportionally the melting curve, compared to the curve of pure *A. muricata* DNA. This happens as the presence of increasing quantity of *A. squamosa* into the *A. muricata* DNA. Real-time PCR and HRM analysis using the *rbcL* primers revealed the levels of substitution or adulteration in the three tested products (P3, P7 and P8) (Fig. 4b). It is indicated that there is at least 12% of *A. squamosa* in P3, 3% of *A. squamosa* in P7 and 25% of *A. squamosa* in P8 with >95% confidence (Fig. 4b). It is therefore undeniable that the *A. muricata* products sold on market are substituted with the inferior or superficially similar species which may or may not have any thereupatic potential. In this case, the products might be adulterated to increase the quantity and to make more profit. All three adulterated products (P3, P7 and P8) were purchased from local producers and thus there is no proper packaging or labeling of the products. In contrast, other tested products (P1-P2, P4-P6 and P9-P11) were purchased from drug stores or supermarkets showing no substitution or adulteration. The results here are similar to those from previous studies of different herbal species^32–35^.

## Conclusion

Based on the forecast *in silico* analysis in finding suitable DNA barcode region for the HRM analysis, there is almost guaranteed success of using Bar-HRM approach for detection of adulteration or substitution in herbal product. Here, the *rbcL* region is the most suitable for discriminating between *Annona* plant species and the Bar-HRM method using *rbcL* primers can be used to investigate the *A. muricata* products sold in processed form such as in tea bags. The relatively high cost is a result of the high market demand of *A. muricata* products and it could be one of the reasons of adulteration found in this study.

## Methods

### Collection of plants samples and tested commercial products

Leaves of the reference plant species including *A. muricata* (soursop), *A. squamosa* (custard apple), and *A. heterophyllus* (jackfruit) were collected in areas of Chiang Mai, Bangkok and Lampoon provinces, Thailand by a taxonomic expert from Chiang Mai University. Eight commercial products, clearly labelled as *A. muricata* (P1-P2, P4-6 and P9-P11) were bought from drug stores or supermarkets and three products (P3 and P7-P8) were bought from local producers and were then used in the adulteration/substitution test.

### Data mining, sequence analyses and simulated high resolution melting (HRM) analysis

The DNA barcode sequences of plant species in genus *Annona* were extracted from GenBank on NCBI (National Center for Biotechnology Information) website using the keyword “*Annona* and each selected barcode region” (*matK*, *rbcL* and *trnL*). Sequence alignment and analysis were done using the MEGA 6 program^36^. The following characteristics were recorded: conserved site (%), variable site (%), average GC content, conserved forward primer/total (%) and conserved reverse primer/total (%). The suitable regions were then used in simulated analysis by uMelt^SM^ application to predict fluorescent high-resolution DNA melting curves of PCR products^37^.

### DNA extraction

The plant material (of both fresh leaves and tea products) was ground with liquid nitrogen, and 100 mg of fine powder was then used for DNA extraction with the Nucleospin Plant® II kit (Macherey-Nagel, Germany) following the manufacturer’s instructions. DNA concentrations of all samples were adjusted equally (20 ng/µL). The DNA was stored at −20 °C for further use.

### Real-time PCR amplification and high resolution melting (HRM) analysis

In order to distinguish the tested plant species, it was necessary to determine the characteristic melting temperature (T_m_) for each sample. Then, PCR amplification, DNA melting, and endpoint fluorescence level acquiring PCR amplifications were performed in a total volume of 20 µL on a Rotor-Gene Q. The reaction mixture contained 20 ng genomic DNA, 10 µL of MeltDoctor™ HRM Master Mix (Applied Biosystems, California, USA), 0.2 µL of 10 mM *rbcL*_F 5′-GGTACATGGACAACTGTGTGGA-3′ and *rbcL*_R 5′-ACAGAACCTTCTTCAAAAAGGTCTA-3′ primers^28^. The real-time PCR reaction conditions are as follows; an initial denaturing step at 95 °C for 5 min followed by 35 cycles of 95 °C for 30 s, 57 °C for 30 s, and 72 °C for 20 s. Subsequently, the PCR amplicons were denatured for HRM at 95 °C for 15 s, and then annealed at 50 °C for 15 s to form random DNA duplexes. Melting curves were generated after the last extension step. The temperature increased from 55 to 95 °C, at 0.1 °C/s. Each species was set as a ‘genotype’ (reference species including *A. muricata*, *A. squamosa*, and *A. heterophyllus*) and the average HRM genotype confidence percentages (GCPs) for the replicates were recorded. The means of the confidence percentage of the species replicates assigned to a representative genotype, together with the standard deviation, were generated.

### Adulteration/Substitution in soursop commercial product test

Eight soursop (*A. Muricata*) tea products (P1-P2, P4-6 and P9-P11) purchased from drug stores or supermarkets and three (*A. Muricata*) tea products (P3 and P7-P8) purchased from local producers were used in the detection of authentication test. DNA isolation and HRM analysis were performed according to the description in the above sections (DNA extraction and Real-time PCR amplification and high resolution melting (HRM) analysis).

## Electronic supplementary material

Supplementary Data 1

Supplementary Data 2
