# Supplementary material for: Bar-HRM for authenticating soursop (Annona muricata) tea
Source: Sci Rep. 2018 Aug 23;8:12666. doi: 10.1038/s41598-018-31127-9 (PMC6107521; doi:10.1038/s41598-018-31127-9)
Supplement: Supplementary file 1 — Supplementary Data 1 [file 41598_2018_31127_MOESM1_ESM.docx]

**Bar-HRM for authenticating soursop (*Annona muricata*) tea**

**Maslin Osathanunkul^1, 2*^**

^1^Department of Biology, Faculty of Science, Chiang Mai University, Chiang Mai, Thailand

^2^Center of Excellence in Bioresources for Agriculture, Industry and Medicine, Chiang Mai University

*Corresponding author: Maslin Osathanunkul, Department of Biology, Faculty of Science, Chiang Mai University, Chiang Mai 50200, Thailand.

E-mail: omaslin@gmail.com. Phone: +66 53 943348. Fax: +66 53 892259

**Supplementary Data 1.** *Annona* sequences of *matK*, *rbcL*, *psbA-trnH* and *trnL* were retrieved from GenBank (NCBI) for each of the species with accession number.

| **Scientific name** | **Accession number (NCBI)** | | | |
| --- | --- | --- | --- | --- |
|  | *matK* | *psbA-trnH* | *rbcL* | *trnL* |
| *Annona acuminata* | GQ981934 | GQ982146 | GQ981664 | - |
| *Annona amazonica* | - | - | EU420853 | EU420836 |
| *Annona bicolor* | - | - | EU420854 | EU420837 |
| *Annona cherimola* | JX495669 | - | JX571777 | - |
| *Annona cornifolia* | - | - | EU420855 | - |
| *Annona deminuta* | - | - | EU420857 | EU420839 |
| *Annona dumetorum* | GQ139704 | - | EU420856 | EU420838, GQ139879 |
| *Annona deceptrix* | - | - | AY841595 | AY841672 |
| *Annona glabra* | GQ139717 | DQ125116 | AY841596 | GQ139891 |
| *Annona holosericea* | - | HG963751 | EU420858 | - |
| *Annona hypoglauca* | - | - | EU420859 | - |
| *Annona montana* | KJ012463 | KJ426607 | EU420860 | - |
| *Annona muricata* | AY743478 | HG963785 | AY743440 | AY743459 |
| *Annona oligocarpa* | - | - | EU420861 | - |
| *Annona prevostiae* | JQ626342 | - | JQ625732 | - |
| *Annona pruinosa* | - | - | EU420862 | EU420844 |
| *Annona purpurea* | JQ586490 | HG963551 | JQ590160 | - |
| *Annona reticulata* | JQ586491-93 | HG963849 | EU420863 | - |
| *Annona rugulosa* | JX880394 | - | JX880395 | - |
| *Annona scandens* | - | - | EU420864 | - |
| *Annona sclerophylla* | GQ139718 | - | - | GQ139892 |
| *Annona senegalensis* | JF270644 | - | AY841597 | AY841674 |
| *Annona symphyocarpa* | - | - | EU420866 | - |
| *Annona spraguei* | GQ981935 | GQ982147 | GQ981665 | - |
| *Annona squamosa* | EU715064 | EU715086 | EU420865 | - |
| *Annona urbaiana* | - | - | EU420867 | EU420849 |
