# Supplementary material for: Bar-HRM for authenticating soursop (Annona muricata) tea
Source: Sci Rep. 2018 Aug 23;8:12666. doi: 10.1038/s41598-018-31127-9 (PMC6107521; doi:10.1038/s41598-018-31127-9)
Supplement: Supplementary file 2 — Supplementary Data 2 [file 41598_2018_31127_MOESM2_ESM.docx]

**Bar-HRM for authenticating soursop (*Annona muricata*) tea**

**Maslin Osathanunkul^1, 2*^**

^1^Department of Biology, Faculty of Science, Chiang Mai University, Chiang Mai, Thailand

^2^Center of Excellence in Bioresources for Agriculture, Industry and Medicine, Chiang Mai University

*Corresponding author: Maslin Osathanunkul, Department of Biology, Faculty of Science, Chiang Mai University, Chiang Mai 50200, Thailand.

E-mail: omaslin@gmail.com. Phone: +66 53 943348. Fax: +66 53 892259

Supplementary Data 2. Differences of *rbcL* sequnces of *Annona* and related species. The number of base sites is 149. Base site homology between the related species and *Annona muricata* is shown as a dot

| **Accession number_Species** | ***rbcL* sequence** |
| --- | --- |
| AY743440_*Annona muricata* | ●●● ●●● ●●● ●●● ●●● ●●● ●●● ●●● ●●● ●●● ●●● ●●● ●●● ●●● ●●● ●●● ●●● ●●● ●●● ●●● ●●● ●●● ●●● ●●● ●●● |
| EU420865_*Annona squamosa* | ●●● ●●● ●●● ●●● ●●● ●●● ●●● ●●● ●●● ●●● ●●● ●●● ●●● ●●● ●●● ●●● ●●● ●●● ●●● ●●● ●●● ●●● A●● ●●● ●●● |
| JX856635_*Artocarpus heterophyllus* | ●●● ●●● ●●● ●●● ●●T ●●A ●●● ●●T ●●C ●●G ●●● ●●● ●●T ●●● ●●● ●●C ●●● ●●● ●●T ●●● ●●● ●●● A●● ●●● ●●● |
| GQ981664_*Annona acuminata* | ●●● ●●● ●●● ●●● ●●● ●●● ●●● ●●● ●●● ●●● ●●● ●●● ●●● ●●● ●●● ●●● ●●● ●●● ●●● ●●● ●●● ●●● ●●● ●●● ●●● |
| EU420853_*Annona amazonica* | ●●● ●●● ●●● ●●● ●●● ●●● ●●● ●●● ●●● ●●● ●●● ●●● ●●● ●●● ●●● ●●● ●●● ●●● ●●● ●●● ●●● ●●● ●●● ●●● ●●● |
| EU420854_*Annona bicolor* | ●●● ●●● ●●● ●●● ●●● ●●● ●●● ●●● ●●● ●●● ●●● ●●● ●●● ●●● ●●● ●●● ●●● ●●● ●●● ●●● ●●● ●●● A●● ●●● ●●● |
| KM068869_*Annona cherimola* | ●●● ●●● ●●● ●●● ●●● ●●● ●●● ●●● ●●● ●●● ●●● ●●● ●●● ●●● ●●● ●●● ●●● ●●● ●●● ●●● ●●● ●●● A●● ●●● ●●● |
| EU420855_*Annona cornifolia* | ●●● ●●● ●●● ●●● ●●● ●●● ●●● ●●● ●●● ●●● ●●● ●●● ●●● ●●● ●●● ●●● ●●● ●●● ●●● ●●● ●●● ●●● ●●● ●●● ●●● |
| AY841595*_Annona deceptrix* | ●●● ●●● ●●● ●●● ●●● ●●● ●●● ●●● ●●● ●●● ●●● ●●● ●●● ●●● ●●● ●●● ●●● ●●● ●●● ●●● ●●● ●●● ●●A ●●● ●●● |
| AY841596*_Annona glabra* | ●●● ●●● ●●● ●●● ●●● ●●● ●●● ●●● ●●● ●●● ●●● ●●● ●●● ●●● ●●● ●●● ●●● ●●● ●●● ●●● ●●● ●●● ●●● ●●● ●●● |
| EU420858*_Annona holosericea* | ●●● ●●● ●●● ●●● ●●● ●●● ●●● ●●● ●●C ●●● ●●● ●●● ●●● ●●● ●●● ●●● ●●● ●●● ●●● ●●● ●●● ●●● ●●● ●●● ●●● |
| EU420859*_Annona hypoglauca* | ●●● ●●● ●●● ●●● ●●● ●●● ●●● ●●● ●●C ●●● ●●● ●●● ●●● ●●● ●●● ●●● ●●● ●●● ●●● ●●● ●●● ●●● ●●● ●●● ●●● |
| KM068881*_Annona macroprophyllata* | ●●● ●●● ●●● ●●● ●●● ●●● ●●● ●●● ●●● ●●● ●●● ●●● ●●● ●●● ●●● ●●● ●●● ●●● ●●● ●●● ●●● ●●● ●●● ●●● ●●● |
| EU420861*_Annona oligocarpa* | ●●● ●●● ●●● ●●● ●G● ●●● ●●● ●●● ●●● ●●● ●●● ●●● ●●● ●●● ●●● ●●● ●●● ●●● ●●● ●●● ●●● ●●● ●●● ●●T ●●● |
| JQ625732*_Annona prevostiae* | ●●● ●●● ●●● ●●● ●●● ●●● ●●● ●●● ●●● ●●● ●●● ●●● ●●● ●●● ●●● ●●● ●●● ●●● ●●● ●●● ●●● ●●● ●●● ●●● ●●● |
| EU420862*_Annona pruinosa* | ●●● ●●● ●●● ●●● ●●● ●●● ●●● ●●● ●●● ●●● ●●● ●●● ●●● ●●● ●●● ●●● ●●● ●●● ●●● ●●● ●●● ●●● A●● ●●● ●●● |
| EU420863*_Annona reticulata* | ●●● ●●● ●●● ●●● ●●● ●●● ●●● ●●● ●●● ●●● ●●● ●●● ●●● ●●● ●●● ●●● ●●● ●●● ●●● ●●● ●●● ●●● A●● ●●● ●●● |
| JX880395*_Annona rugulosa* | ●●● ●●● ●●● ●●● ●●● ●●● ●●● ●●● ●●● ●●● ●●● ●●● ●●● ●●● ●●● ●●● ●●● ●●● ●●● ●●● ●●● ●●● ●●● ●●● ●●● |
| EU420864*_Annona scandens* | ●●● ●●● ●●● ●●● ●●● ●●● ●●● ●●● ●●C ●●● ●●● ●●● ●●● ●●● ●●● ●●● ●●● ●●● ●●● ●●● ●●● ●●● ●●● ●●● ●●● |
| AY841597*_Annona senegalensis* | ●●● ●●● ●●● ●●● ●●● ●●● ●●● ●●● ●●● ●●● ●●● ●●● ●●● ●●● ●●● ●●● ●●● ●●● ●●● ●●● ●●● ●●● ●●● ●●● ●●● |
| GQ981665*_Annona spraguei* | ●●● ●●● ●●● ●●● ●●● ●●● ●●● ●●● ●●C ●●● ●●● ●●● ●●● ●●● ●●● ●●● ●●● ●●● ●●● ●●● ●●● ●●● ●●● ●●● ●●● |
| KU568002*_Annona stenophylla* | ●●● ●●● ●●● ●●● ●●● ●●● ●●● ●●● ●●● ●●● ●●● ●●● ●●● ●●● ●●● ●●● ●●● ●●● ●●● ●●● ●●● ●●● ●●● ●●● ●●● |
| EU420866*_Annona symphyocarpa* | ●●● ●●● ●●● ●●● ●●● ●●● ●●● ●●● ●●● ●●● ●●● ●●● ●●● ●●● ●●● ●●● ●●● ●●● ●●● ●●● ●●● ●●● ●●● ●●● ●●● |
| EU420867*_Annona urbaniana* | ●●● ●●● ●●● ●●● ●●● ●●● ●●● ●●● ●●● ●●● ●●● ●●● ●●● ●●● ●●● ●●● ●●● ●●● ●●● ●●● ●●● ●●● A●● ●●● ●●● |

Supplementary Data 2. (continue) Differences of *rbcL* sequnces of *Annona* and related species. The number of base sites is 149. Base site homology between the related species and *Annona muricata* is shown as a dot

| **Accession number_Species** | ***rbcL* sequence** |
| --- | --- |
| AY743440_*Annona muricata* | ●●● ●●● ●●● ●●● ●●● ●●● ●●● ●●● ●●● ●●● ●●● ●●● ●●● ●●● ●●● ●●● ●●● ●●● ●●● ●●● ●●● ●●● ●●● ●●● ●● |
| EU420865_*Annona squamosa* | ●●● ●●● ●TC ●●● ●●● ●●● ●●● ●●● ●●● ●●● ●●● ●●● ●●● ●●● ●●● ●●● ●●● ●●● ●●● ●●● ●●● ●●● ●●● ●●● ●● |
| JX856635_*Artocarpus heterophyllus* | ●●C ●●● ●●● ●●● ●●A ●●● ●●● ●●● ●●● ●●● ●●● ●●● ●●● ●●● ●●● ●●C ●●● ●●C ●●● ●●● ●●A ●●A ●●● ●●● ●● |
| GQ981664_*Annona acuminata* | ●●● ●●● ●●C ●●● ●●● ●●● ●●● ●●● ●●● ●●● ●●● ●●● ●●● ●●● ●●● ●●● ●●● ●●● ●●● ●●● ●●● ●●● ●●● ●●● ●● |
| EU420853_*Annona amazonica* | ●●● ●●● ●●C ●●● ●●● ●●● ●●● ●●● ●●● ●●● ●●● ●●● ●●● ●●● ●●● ●●● ●●● ●●● ●●● ●●● ●●● ●●● ●●● ●●● ●● |
| EU420854_*Annona bicolor* | ●●● ●●● ●TC ●●● ●●● ●●● ●●● ●●● ●●● ●●● ●●● ●●● ●●● ●●● ●●● ●●● ●●● ●●● ●●● ●●● ●●● ●●● ●●● ●●● ●● |
| KM068869_*Annona cherimola* | ●●● ●●● ●TC ●●● ●●● ●●● ●●● ●●● ●●● ●●● ●●● ●●● ●●● ●●● ●●● ●●● ●●● ●●● ●●● ●●● ●●● ●●● ●●● ●●● ●● |
| EU420855_*Annona cornifolia* | ●●● ●●● ●●C ●●● ●●● ●●● ●●● ●●● ●●C ●●● ●●● ●●● ●●● ●●● ●●● ●●● ●●● ●●● ●●● ●●● ●●● ●●● ●●● ●●● ●● |
| AY841595*_Annona deceptrix* | ●●C ●●● ●●C ●●● ●●● ●●● ●●● ●●● ●●● ●●● ●●● ●●● ●●● ●●● ●●● ●●● ●●● ●●● ●●● ●●● ●●● ●●● ●●● ●●● ●● |
| AY841596*_Annona glabra* | ●●● ●●● ●●C ●●● ●●● ●●● ●●● ●●● ●●● ●●● ●●● ●●● ●●● ●●● ●●● ●●● ●●● ●●● ●●● ●●● ●●● ●●● ●●● ●●● ●● |
| EU420858*_Annona holosericea* | ●●C ●●● ●●C ●●● ●●● ●●● ●●● ●●● ●●● ●●● ●●● ●●● ●●● ●●● ●●● ●●● ●●● ●●● ●●● ●●● ●●● ●●● ●●● ●●● ●● |
| EU420859*_Annona hypoglauca* | ●●C ●●● ●●C ●●● ●●● ●●● ●●● ●●● ●●● ●●● ●●● ●●● ●●● ●●● ●●● ●●● ●●● ●●● ●●● ●●● ●●● ●●● ●●● ●●C ●● |
| KM068881*_Annona macroprophyllata* | ●●● ●●● ●TC ●●● ●●● ●●● ●●● ●●● ●●● ●●● ●●● ●●● ●●● ●●● ●●● ●●● ●●● ●●● ●●● ●●● ●●● ●●● ●●● ●●● ●● |
| EU420861*_Annona oligocarpa* | ●●● ●●● ●●C ●●● ●●● ●●● ●●● ●●● ●●● ●●● ●●● ●●● ●●● ●●● ●●● ●●● ●●● ●●● ●●● ●●● ●●● ●●● ●●● ●●● ●● |
| JQ625732*_Annona prevostiae* | ●●● ●●● ●●C ●●● ●●● ●●● ●●● ●●● ●●● ●●● ●●● ●●● ●●● ●●● ●●● ●●● ●●● ●●● ●●● ●●● ●●● ●●● ●●● ●●● ●● |
| EU420862*_Annona pruinosa* | ●●● ●●● ●TC ●●● ●●● ●●● ●●● ●●● ●●● ●●● ●●● ●●● ●●● ●●● ●●● ●●● ●●● ●●● ●●● ●●● ●●● ●●● ●●● ●●● ●● |
| EU420863*_Annona reticulata* | ●●● ●●● ●TC ●●● ●●● ●●● ●●● ●●● ●●● ●●● ●●● ●●● ●●● ●●● ●●● ●●● ●●● ●●● ●●● ●●● ●●● ●●● ●●● ●●● ●● |
| JX880395*_Annona rugulosa* | ●●● ●●● ●●C ●●● ●●● ●●● ●●● ●●● ●●● ●●● ●●● ●●● ●●● ●●● ●●● ●●● ●●● ●●● ●●● ●●● ●●● ●●● ●●● ●●● ●● |
| EU420864*_Annona scandens* | ●●C ●●● ●●C ●●● ●●● ●●● ●●● ●●● ●●● ●●● ●●● ●●● ●●● ●●● ●●● ●●● ●●● ●●● ●●● ●●● ●●● ●●● ●●● ●●C ●● |
| AY841597*_Annona senegalensis* | ●●● ●●● ●●C ●●● ●●● ●●● ●●● ●●● ●●● ●●● ●●● ●●● ●●● ●●● ●●● ●●● ●●● ●●● ●●● ●●● ●●● ●●● ●●● ●●● ●● |
| GQ981665*_Annona spraguei* | ●●C ●●● ●●C ●●● ●●● ●●● ●●● ●●● ●●● ●●● ●●● ●●● ●●● ●●● ●●● ●●● ●●● ●●● ●●● ●●● ●●● ●●● ●●● ●●● ●● |
| KU568002*_Annona stenophylla* | ●●● ●●● ●●C ●●● ●●● ●●● ●●● ●●● ●●● ●●● ●●● ●●● ●●● ●●● ●●● ●●● ●●● ●●● ●●● ●●● ●●● ●●● ●●● ●●● ●● |
| EU420866*_Annona symphyocarpa* | ●●● ●●● ●●C ●●● ●●● ●●● ●●● ●●● ●●● ●●● ●●● ●●● ●●● ●●● ●●● ●●● ●●● ●●● ●●● ●●● ●●● ●●● ●●● ●●● ●● |
| EU420867*_Annona urbaniana* | ●●● ●●● ●TC ●●● ●●● ●●● ●●● ●●● ●●● ●●● ●●● ●●● ●●● ●●● ●●● ●●● ●●● ●●● ●●● ●●● ●●● ●●● ●●● ●●● ●● |
